# The ubiquitination code: a signalling problem

**DOI:** 10.1186/1747-1028-2-11

**Published:** 2007-03-13

**Authors:** Tanja Woelk, Sara Sigismund, Lorenza Penengo, Simona Polo

**Affiliations:** 1 IFOM, Istituto FIRC di Oncologia Molecolare, Via Adamello 16, 20139, Milan, Italy

## Abstract

Ubiquitin is a highly versatile post-translational modification that controls virtually all types of cellular events. Over the past ten years we have learned that diverse forms of ubiquitin modifications and of ubiquitin binding modules co-exist in the cell, giving rise to complex networks of protein:protein interactions. A central problem that continues to puzzle ubiquitinologists is how cells translate this myriad of stimuli into highly specific responses. This is a classical signalling problem. Here, we draw parallels with the phosphorylation signalling pathway and we discuss the expanding repertoire of ubiquitin signals, signal tranducers and signalling-regulated E3 enzymes. We examine recent advances in the field, including a new mechanism of regulation of E3 ligases that relies on ubiquitination.

## Background

Cells use a system of post-translational protein modifications to generate and transmit signals in almost every pathway. One of them is ubiquitination. When appended to target proteins ubiquitin (Ub) can affect their localization, activity, structure and interaction partners. Similarly to the phospho-based signal, the ubiquitin signal is recognized by specific domains (Ubiquitin Binding Domains, UBDs), carried by proteins (Ub receptors) that represent signal transducers. More parallels can be drawn with phosphorylation: ubiquitination is reversible thanks to deubiquitinating enzymes (DUBs) and it is inducible by various stimuli. Compared to phosphorylation, the signal generated by the 76-residue Ub protein is certainly more complex. Ub can be appended as a monomer or as isopeptide-linked polymers named polyubiquitin chains. The existence of a vast array of Ub signals raises the question of how the specificity of signal recognition might be achieved. Indeed, the molecular requirements for recognition of the various known Ub signals are poorly understood and form a central question in the field.

Here, we will discuss recent studies that shed new light on the Ub code and that regard the topology of Ub signals, the identification of new UBDs and the regulation of the critical enzymes of the signalling cascade, namely, the E3 ligases.

## Ubiquitin chain complexity: generation of signalling diversity

**Figure 1:**
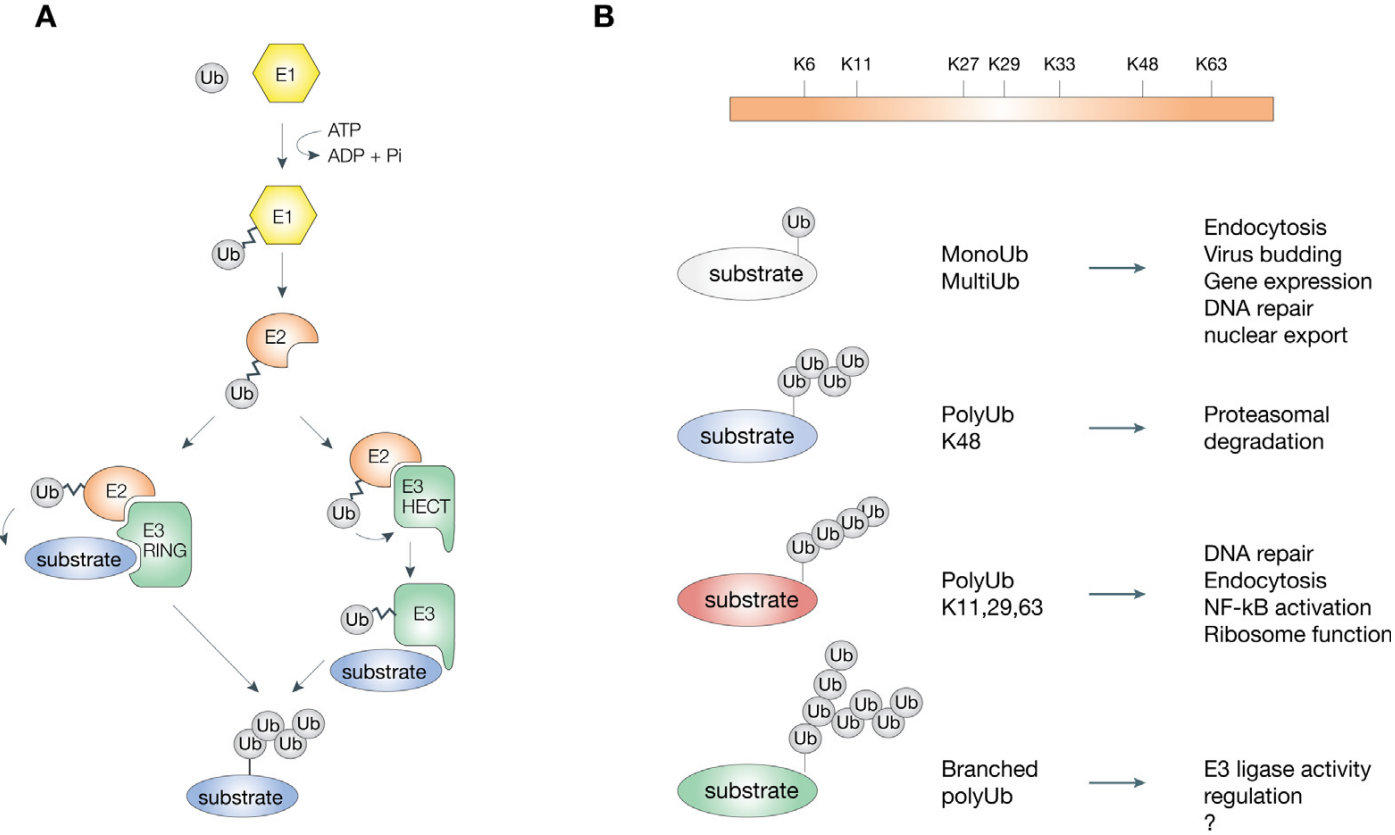
**The ubiquitin pathway**. A) Schematic representation of the ubiquitination process. A hierarchical set of three types of enzyme is required for substrate ubiquitination: ubiquitin-activating (E1), ubiquitin-conjugating (E2) and ubiquitin-protein ligase (E3) enzymes. The two major classes of E3 ligases are depicted. B) Schematic representation of the different Ub modifications with their functional roles. The question mark indicates that the functions of branched chains are largely unknown.

Substrate proteins can be modified by Ub in different ways (Figure 1). Monoubiquitination is the attachment of one Ub moiety to a single lysine residue. This modification is a reversible, non-proteolytic signal involved in endocytosis, endosomal sorting, histone regulation, DNA repair, virus budding and nuclear export (reviewed in [1-4]). A variation of this theme occurs when several lysine residues of a substrate are modified by a single Ub molecule, giving rise to multiple monoubiquitination that plays a role in receptor internalization and endocytosis [5-7].

Ubiquitin itself contains seven lysine residues that can be potentially used as acceptors for the attachment of other Ub molecules, allowing the formation of different types of Ub chains (polyubiquitination, Figure 1). Indeed, it has recently been shown that all seven lysine residues are used *in vivo *for chain formation [8,9]. However, it is currently not known if all of the linkages have a specific function.

Lysine linkage is a central issue, since different polyUb chains contribute to the generation of diversity in Ub-dependent signalling. The first genetic evidence that structurally different chains might represent functionally distinct signals, came from a study showing that yeast cells expressing Ub-K63R are defective in DNA repair, but still proteolytically competent [10]. The simplest idea to explain chain recognition is based on possible different topologies adopted by different chain types. Such a concept would help to rationalize how cells interpret the diversity of Ub signals and translate them into specific biological responses.

The best-studied examples are chains of four or more Ub moieties linked through lys48. This form of chain targets proteins for degradation via the 26S proteasome [11,12]. Interestingly, NMR and crystallographic studies have shown that lys48-linked chains adopt a closed conformation, whereby hydrophobic residues of two adjacent Ub molecules are exposed at the interface and contact each other [13-16]. Such structural attributes appear to be required for the recognition by the proteasomal subunits [12,17].

Ub-chains formed through lys63 have also been subjected to intense scrutiny. Similarly to monoubiquitin, such chains generate a non-proteolytic signal involved in DNA repair, transcriptional regulation, endocytosis and activation of protein kinases [18-20]. NMR data have confirmed the existence of a conformational difference between lys48- and lys63-linked chains, with the latter adopting an extended, linear conformation of Ub units arranged head to tail [21,22]. This type of structure suggests that lys63 chains might be recognized as a signal topologically similar to monoubiquitin. Indeed, while monoUb is sufficient to stimulate rapid internalization for the majority of yeast proteins [23,24], lys63-linked chains are required for maximal internalization rates of a subset of nutrient permeases [18,25]. A similar behaviour has been observed in mammalian cells. MHC class I molecules [26] and nerve growth factor receptor TrkA [27] require lys63 chains for internalization, while EGFR is modified by both monoUb and lys63 chains [5,7].

Much less is known about the precise function and topology of chains that are linked through lys6, lys11, lys27, lys29 and lys33 [3,12,20] and structural analysis are needed to understand if these chains also have peculiar conformational properties.

New pioneering studies demonstrating that atypical, mixed polyUb chains can be formed, point to an additional level of complexity in the Ub system. Several groups have reported the identification of branched chains containing different types of linkages, but their functions still remain unclear [8,28-30]. These findings are based exclusively on *in vitro *reconstituted systems, often performed using lysine-to-arginine mutant forms of Ub. Despite the proved utility of these molecular approaches they remain indirect and susceptible of criticisms. Unfortunately, this is our resolution limit at present. More work is required to understand the relevance of these different lysine linkages *in vivo *and, in this respect, the development of novel diagnostic tools, such as linkage-specific antibodies or innovative mass spectrometry strategies, would be of great help.

## Ub and endocytosis: EGFR as a case study

Receptor Tyrosine Kinases (RTKs) control different biological processes including cell proliferation, differentiation and survival [31]. The prototype of RTKs is the Epidermal Growth Factor Receptor (EGFR), which plays critical roles in physiological and pathological processes in epithelial cells [31]. Once activated by its cognate ligand, the EGFR undergoes dimerization and trans-phosphorylation at different tyrosines, which serve as docking sites for several signalling/adaptor molecules [32]. One of these is the E3 ligase Cbl, which mediates ubiquitination of the receptor [33,34]. In a first set of studies, EGFR was shown to be primarily modified by multiple monoubiquitination *in vivo *[5,6] and a single Ub moiety was demonstrated to be sufficient to drive receptor internalization [5]. More recently, a challenging study based on a mass spectrometry approach has shown that, in addition to multiple monoubiquitination, the EGFR is modified by short lys63-linked chains within the kinase domain [7]. Although we cannot exclude that these two EGFR modifications may play distinct role in endocytosis, the structural data discussed previously argue for a topologically-related signal. The mechanism through which lys63-linked chains could further stimulate internalization is not known at the moment. The simplest explanation is that Ub lys63 chains might work as a tandem monoUb signal, increasing the affinity for Ub receptors that operate at the various stations along the endocytic pathway, but other explanations are also possible (for detailed examples see [4]).

**Figure 2:**
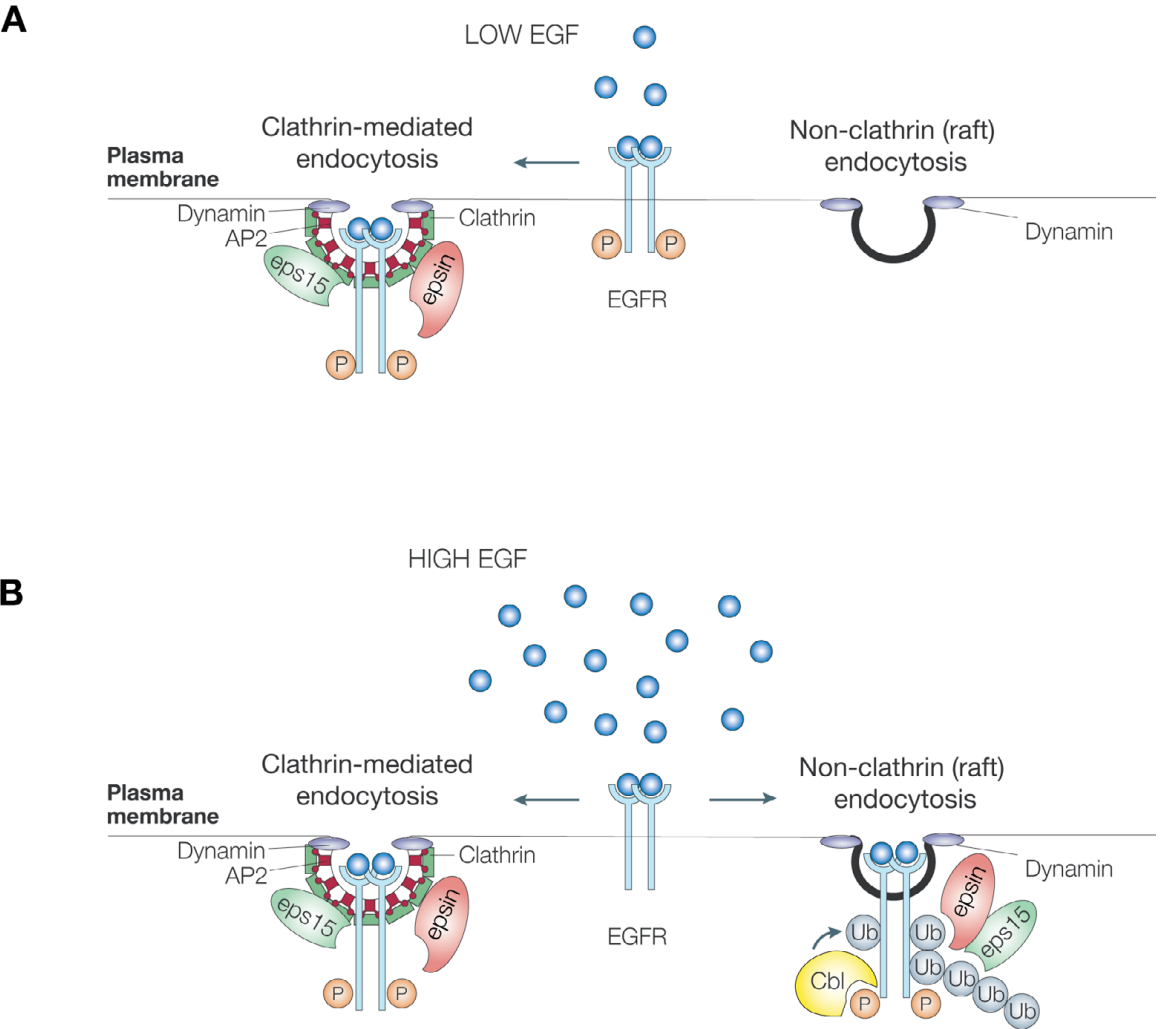
**Dose-dependent entry routes for the EGFR**. A) At low doses of EGF, EGFR is mainly internalized through clathrin pathway (CME, clathrin-mediated endocytosis). In this condition, ubiquitination of the receptor is not required for its internalization. B) At higher doses of EGF, CME is equally active but a significant part of EGFR internalization proceeds also to an alternative pathway (NCE, non-clathrin endocytosis). The exact nature, and the molecular determinants, of this "alternative" pathway are still controversial. Essentially NCE is defined by its insensitivity to functional ablation (KD) of clathrin and sensitivity to cholesterol-interfering drugs, like filipin. Hence, its definition as a "raft-dependent pathway". Internalization of EGFR through NCE requires receptor ubiquitination. Multiple roles of Eps15/R and epsin in the different internalization pathways are also shown (see main text for detailed explanations).

The best characterized role for receptor ubiquitination is to negatively regulate signalling by targeting receptors for lysosomal degradation (reviewed in [35-37]). The requirement for Ub at the initial step of internalization of EGFR was rather controversial [38-41]. The studies we conducted under physiological conditions helped to resolve this issue and unveiled the importance of ligand concentration in the internalization process [42]. We found that the receptor uses different pathways depending on its ubiquitination state, which is a direct consequence of the amount of EGF used to stimulate it. Indeed, at low doses of EGF (1.5 ng/ml), only clathrin-dependent endocytosis is active. For this pathway, receptor ubiquitination is not necessary. Accordingly, mutations, either targeting the tyrosine docking-site for Cbl or the ubiquitinated lysines, resulting in a Ub-defective receptor, abrogate receptor down-regulation but not its internalization via the clathrin pathway [7,39,40,42]. At higher, but still physiologically relevant, EGF concentration (20 ng/ml), an additional clathrin-independent, raft-dependent pathway comes into play, concomitantly with ubiquitination of the receptor. Experiments with EGFR mutants that can no longer be ubiquitinated or engineered to have Ub as the only intracellular signal (EGFR-Ub chimera) confirm that Ub is the major device that directs the receptor to the raft pathway [42]. A schematic view of the different entry routes of EGFR in HeLa cells and their regulation as a function of EGF dose is depicted in Figure 2.

The existence of distinct, highly regulated internalization pathways, raises the possibility that they might be involved in the generation of signalling diversity, by targeting the receptor to different fates, as was shown in the case of the TGFβ receptor [43]. To date, the impact of the two pathways on the final biological outcomes of EGFR signalling is not known and remains a challenging question. We recently found that while the raft-dependent pathway is preferentially associated with EGFR degradation, clathrin-dependent internalization appears to contribute primarily to EGFR recycling and signalling (SS and SP, unpublished).

Three adaptors, eps15, eps15R and epsin, are recruited to the EGFR at the plasma membrane upon ligand activation. These proteins have been traditionally linked to clathrin-dependent endocytosis, due to their multiple interactions with the clathrin apparatus [44-49]. Recent evidence, while confirming their involvement in the clathrin pathway with an optimizing function, point to a previously undiscovered requirement for these adaptors in targeting Ub-conjugated cargo to clathrin-independent endocytosis [42,50]. Indeed, both eps15/R and epsin present single or multiple Ub interacting motifs (UIMs), through which they recognize the ubiquitinated receptor and mediate its clathrin-independent internalization [42,51,52]. Hence, it is becoming clear that these endocytic adaptors may play multiple roles in different endocytic pathways, depending on which signal the cargo protein presents and which endocytic route cargo uses.

Interestingly, these proteins are themselves modified by monoUb in ligand-dependent manner [52,53]. By creating additional surfaces of interaction, monoubiquitination of adaptors could contribute to signal amplification [1,4,53], as exemplified by findings that growth hormone receptor internalization is crucially dependent on functional Ub machinery, but does not require receptor ubiquitination [54]. In this and other cases [55,56], monoubiquitinated adaptors might be responsible for sorting of receptors along the endocytic pathway. An alternative possibility was recently proposed by Dikic and colleagues [57]. In this model monoubiquitination of the adaptor leads to an intramolecular interaction mediated by the UBD that folds back on the Ub appended in *cis*. This switch-off mechanism allows the detachment of the adaptor from the ubiquitinated cargos [4,57]. At least for eps15, evidence is accumulating in our laboratory that argues against an intramolecular inhibitory interaction mediated by the UIM (TW and SP, unpublished). It should be mentioned that the precise role of monoubiquitination could be distinct in the different adaptors and that the present knowledge does not allow generalization of one specific concept.

In conclusion, the emerging picture indicates that Ub can regulate EGFR endocytosis and signalling in several ways. On one hand, by targeting the receptor, Ub mediates its internalization and degradation, leading to signal termination. On the other hand, by targeting the endocytic machinery, Ub might contribute to the "endocytic" regulation of signalling in time and space. The combination of these Ub-based regulatory mechanisms together with others, such as phosphorylation, contribute to generate the wide variety of biological responses downstream of the EGFR.

## UBD specificity: recognition of signalling diversity

The identification of the first Ub binding modules has set the stage for the discovery of a Ub-based network of protein:protein interactions [1]. Proteins carrying UBDs represent the downstream effectors able to recognize and further transmit the Ub signal. In terms of structure and affinity characteristic UBDs have been amply and excellently reviewed [53,58,59]. Here, we concentrate on possible mechanisms of linkage-specific recognition.

Despite the increased number of high-resolution structural studies, no single domain can be accurately referred to as mono- or polyubiquitin-specific binding domain. In a systematic effort to define the factors responsible for linkage-specific recognition, Pickart and co-workers examined the Ub interaction properties of 30 distinct UBA (ubiquitin associated) domains and sorted them in four different classes [60]. Class 1 domains, which include the UBA domains from Rad23 and hHR23A, selectively recognize lys48-linked chains. This preference is consistent with the role of Rad23 as a modulator of proteasome activity, although the UBA might be not essential for this function. Class 2 domains preferentially bind lys63-linked chains. Among others, this group includes the UBA from Ede1, the yeast homolog of eps15, which mediates protein trafficking typically associated with monoUb or lys63-linked chains. Class 3 domains comprise many UBAs that do not bind to Ub at all. Although it cannot be excluded that they recognize Ub chains different from the ones used or other Ub-like protein modifiers, these results underline the need for experimental validation of bioinformatically identified UBD. The last class of UBA domains bind to polyUb chains without any linkage specificity. Unfortunately, while the classification was certainly useful, it did not support any distinction based on primary sequence comparison, leaving the molecular basis for linkage-specific discrimination still unclear. It should be noticed that the preference observed for an isolated UBD could be different from the one observed for the full-length protein. *In vivo *subcellular localization as well as structural constraints might strongly influence the type of Ub that Ub receptors are programmed to bind.

In some cases, preferences for lys48-linked chains can be explained by the ability of a single UBD to interact with two Ub moieties in 1:2 stoichiometry. NMR studies have revealed the modes of interaction of the UBA domains of hHRD23A and of Mud1 with two Ub molecules linked via lys48 [22,61]. In both cases, the UBA appears to insert into the di-ubiquitin making contact with both Ub moieties. Interestingly, the hHRD23A UBA2 domain seems to bind the lys48-linkage directly [22]. The recent characterization of the structure of the UIM (Ub-interacting motif) of Hrs in complex with Ub revealed a comparable mode of interaction [62]. Hrs is a key protein involved in the sorting of ubiquitinated cargos. The authors showed that UIM of Hrs is capable of binding two Ub molecules at the same time through two interaction surfaces embedded on the opposite sides of the same helix. Based on sequence comparison, they identified a new subclass of UIMs that was named DUIM (double-sided UIM). In addition to increased avidity, a possible intriguing feature of these domains is the potential for binding to topologically distinct chains, although this has not been experimentally tested.

The protein NEMO, an essential regulator of NF-kB activation, represents an interesting case of chain-specific recognition [63,64]. Pull-down experiments have demonstrated that the region encompassing the second coiled-coil and the leucine zipper motifs is able to bind lys63-linked chains, but not chains linked through lys48 [63,64]. One physiological substrate of NEMO is the receptor-interacting protein, RIP, which is indeed lys63-ubiquitinated [65]. It has been proposed that NEMO protects RIP from the proteasomal degradation by preventing the conversion of lys63-linked to lys48-linked polyubiquitination promoted by the A20 protein [63,65].

Despite the high degree of conservation of the entire Ub molecule throughout evolution, the majority of Ub receptors contact the hydrophobic face of Ub, centred on Ile44. A pioneering study performed in S. cerevisiae has identified other functionally relevant regions in Ub molecules [66]. Recently three novel UBDs were reported to recognize alternative surfaces of Ub. Using a two-hybrid screening approach with a mutated version of Ub (Ile44Ala) Dikic and collegues identified the UBM (Ub binding motif) present in Y-family translesion synthesis (TLS) polymerases [67]. NMR studies showed that the UBM recognizes a Ub surface around Leu8, that is near, but not overlapping with, Ile44. The presence of this domain allows TLS polymerases to interact with ubiquitinated PCNA after DNA damage [67,68].

**Figure 3:**
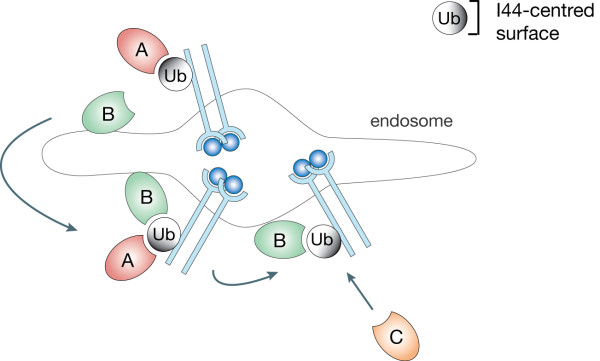
**"Hand-off" mechanism model for ubiquitinated cargos**. Ub receptors (A and C), recognizing the hydrophobic patch centred on Ile44 (depicted in black), are intercalated with others (B), recognizing different region of Ub (depicted in white). Rabex-5, through ZnF_A20 domain, recognizes the polar patch centred on Asp58 and represents an example of Ub receptors B. Endosome is depicted as prototype of trafficking organelles.

The second non-canonical Ub binder is the ZnF_A20 domain present in Rabex-5, a Rab5 guanine nucleotide exchange factor. Crystal structures have revealed that the ZnF_A20 makes contacts with a polar patch centred on Asp58, which is completely displaced from Ile44 [69,70]. Functionally, we have demonstrated that the ZnF_A20 domain is required, together with the adjacent MIU (motif interacting with Ub) for the binding of Rabex-5 to the activated and ubiquitinated EGFR [69]. The discovery that a single Ub moiety can simultaneously engage two different partners increases the complexity of Ub-based network and provides interesting outlooks. For instance, it can explain how Ub receptors promote trafficking of ubiquitinated cargos along the endocytic route. The "hand-off" mechanism previously proposed [53] is difficult to hypothesize if the sole interaction surface on Ub is around Ile44. If, instead, we envision a scenario in which Ub receptors that recognize non-canonical surfaces of Ub are intercalated with Ile44-specific Ub receptors, the hand-off mechanism of trafficking of monoubiquitinated cargos becomes plausible (Figure 3).

The third example of an alternative Ub recognition site is represented by the UBD present in the deubiquitinating enzyme isopeptidase T (IsoT or USP5), named ZnF_UBP. This domain contains a unique, deep tunnel-like binding pocket which can accomodate the C-terminus diglycine motif of Ub [71]. In addition to this extensive interaction, ZnF_UBP also binds to Ub around Ile36, while no contacts with the Ile44 patch are evident. Functionally, IsoT is responsible for the hydrolysis of unanchored polyUb chains. It releases one Ub at a time, starting from the proximal end of the chain [71]. Mutations in the ZnF_UBP domain demonstrate that it is required for optimal catalytic activation of IsoT. Given the different conformations of adopted different types of polyUb chains, it is unclear how IsoT can act on all of them. A speculative hypothesis implicates the high flexibility of the C terminus of Ub that can easily rotate in order to position the scissile bond of alternatively linked polyubiquitin chains near the catalytic site. The proximal Ub in the chain is immobilized via the insertion of its C-terminus diglycine motif in the deep invagination of the ZnF_UBP. Since ZnF_UBP does not contact any lysine residues that could possibly be involved in chain linkage, the chain could project out from the proximal Ub in any direction, thus enabling IsoT to trim any type of Ub chain.

## E3 ligases: signalling-regulated enzymes

Ubiquitination is often induced by extracellular stimuli and substrates are not constitutively recognized. Thus, the activity of the ubiquitin system needs to be rapidly and tightly modulated in order to gain specificity. This could be achieved promoting or inhibiting substrate recognition and/or modifying the catalytic activity of the conjugating enzymes [72].

Regulation can occur via the subcellular localization of the enzymes or of the substrates or via the binding to "auxiliary" proteins. In the case of TGF-β signalling, Smad7 acts as an adaptor protein and recruits the Smurf E3 ligases to the TGF-β receptor [73]. In addition, Smad7 is able to stimulate the catalytic activity of Smurf2 by recruiting and positioning the E2, UbcH7, in close proximity to the HECT domain of Smurf2 [74]. Accessory proteins may also have an inhibitory function, such as the tumor suppressor protein ARF which blocks the E3 ligase activity of Mdm2 leading to p53 stabilization [75].

Regulation can also occur through post-translational modification of the substrate or of a component of the ubiquitin conjugating machinery. Usually, the E3 ligase is the key component of the machinery subject to regulation, although post-translational regulation of E2 enzymes has recently been described [76-79].

## Regulation by phosphorylation

In recent years, it has become clear that Ub regulation is often dependent upon protein phosphorylation [72]. Phosphorylation may play a role in either the recognition of substrate by the E3 ligase or the modulation of E3 enzymatic activity.

Substrate phosphorylation is a common and well-characterized way of creating a binding site for the E3 ligase. Many multisubunit RING E3s, such as Skp1-Cul1-F-box protein (SCF) complexes, target their substrates for proteasomal degradation in a phosphorylation-dependent manner. Phosphorylated substrates are recognized and recruited to the SCF core complex through one of the large family of F-box adaptors proteins [80]. Cbl, a monomeric RING E3 ligase involved in the ubiquitination of the EGFR, binds its substrate through the N-terminal PTP (phosphotyrosine binding) domain only after the receptor is phosphorylated [34]. Phosphorylation might also prevent substrate recognition/interaction by the E3. C-Jun tyrosine phosphorylation within its PPXY motif by c-Abl inhibits binding to its E3 ligase Itch and, as a consequence, prevents ubiquitination and proteasomal degradation of c-Jun [81].

On the other side, E3 phosphorylation is now accepted as an E3 regulatory mechanism. Its molecular consequences are only beginning to be explored. Phosphorylation of an E3 can generally inhibit or activate the enzymatic activity towards specific substrates (for detailed examples see [72]). In the case of negative regulation, recognition of the substrate is generally impaired. In addition, E3 activity is often shifted to self-ubiquitination or to another substrate [82-84]. Alterations of the subcellular localization have been described, as well [84-86].

Recently, Karin and colleagues have elucidated the mechanism of activation for the JunB E3 ligase Itch, which is critically important in T helper cell differentiation [87]. After T cell receptor engagement, Itch undergoes JNK1-mediated phosphorylation that greatly enhances its enzymatic activity. The authors found that under non-stimulated conditions, Itch is inactive due to an intramolecular interaction between its WW and HECT domains. After T-cell stimulation, Itch is phosphorylated on three key residues (S199, T222, S232) within the proline-rich region. This phosphorylation event weakens the intramolecular interaction, most likely through electrostatic repulsions, leading to a conformational change in the WW domain. This conformational change releases the self-inhibitory interaction, thus dramatically increasing the E3-ligase activity [87]. In addition to undergoing serine/threonine phosphorylation, Itch is tyrosine phosphorylated by Fyn, a member of the Src family kinases, after T-cell stimulation [88]. Various observations suggest a negative regulation of Itch by tyrosine phosphorylation [88]. The mechanism by which this happens is unclear, but it has been proposed that tyrosine phosphorylation might sterically inhibit the interaction between Itch and JunB [88]. Thus, Itch-mediated JunB degradation seems to be tightly regulated by counterbalancing serine/threonine and tyrosine phosphorylation. It will be interesting to see if similar mechanisms of regulation exist for other types of E3 ligases for which phosphorylation events by various extracellular stimuli have been described [72].

## Regulation by ubiquitination

E3 ligases can be regulated by ubiquitination. The simplest possibility is that E3 ligases are modified by a polyubiquitin chain linked through lys48 and targeted for proteasomal degradation. RING finger-containing E3s often catalyze their own polyubiquitination, in addition to polyubiquitination of their substrates [89]. HECT-E3s are also capable of self-ubiquitination [53]. Self-ubiquitination is a well accepted auto-regulatory mechanism which controls E3 ligase levels by degradation [90-92]. An additional level of regulation can be achieved through different E3 ligases acting on one other. This concept might help rationalize why, in some cases, different E3 ligases are found in the same complex, such as, for instance, Cbl and Nedd4 or AIP4 [93,94].

Two recent papers have shown a non-destructive regulatory function for Ub modifications of E3 ligases. In both cases, the Ub modification enables the E3 ligases to promote monoubiquitination of their substrates [30,95]. Our group has demonstrated that the ubiquitination of the HECT-type E3 ligase Nedd4 is required for recognition of a specific substrate, eps15 and confers to the enzyme the ability to execute coupled monoubiquitination [95]. Eps15 is one of the Ub receptors, already described, involved in EGFR internalization. Like many other Ub receptors, eps15 becomes monoubiquitinated upon EGF stimulation by a process known as coupled monoubiquitination, which requires the presence of an intact UBD [52,53]. Nedd4 is the *bona fide *E3 ligase for eps15 but no stable interaction between these two proteins has been detected [52]. Since the UIM:Ub interaction is required for eps15 ubiquitination, we hypothesised that the UIM of eps15 may bind a ubiquitinated form of Nedd4. Two possibilities were envisioned. In the first one that we called "E3-thiolester model", the UIM would bind to the Ub linked through the thiolesther bond at the Nedd4 catalytic cysteine. The same Ub would be then directly transferred to the substrate. In the second model, called "E3-isopeptide model", the UIM would bind to a covalently ubiquitinated Nedd4 and then the catalytic cysteine would transfer another Ub to the substrate. To distinguish between the two models, a key experiment based on the capacity of Nedd4 to undergo self-ubiquitination *in vitro *was performed. The "E3-isopeptide model" predicts that increasing the amount of Ub-conjugated Nedd4 in the reaction should then increase monoubiquitination of eps15. Conversely, if the "E3-thiol-ester model" is correct, then eps15 ubiquitination should be indifferent to the status of Nedd4. In agreement with the isopeptide model, we found that ubiquitinated Nedd4 dramatically enhanced the amount of eps15 monoubiquitination with significantly faster kinetics. No differences were observed when a conventional substrate for Nedd4 was used [95]. Thus, ubiquitination of the enzyme enables the E3 to recognize and ubiquitinate an additional substrate without changing its catalytic activity. If this model might be applicable to other UBDs capable of coupled monoubiquitination deserves further investigations.

In a completely different system, Ciechanover and collegues have demonstrated that self-ubiquitination of Ring1B is required for subsequent monoubiquitination of its substrate, H2A [30]. When the ability of Ring1B to undergo self-ubiquitination was analysed, the authors found that the chains generated by the enzyme are atypical, mixed polyUb chains that do not serve as a proteasome targeting signal. With the help of an array of lys-modified Ub molecules, the authors convincingly proved that three lysines, namely lys6, lys27 and lys48, should be present on the same ubiquitin molecule to achieve full self-ubiquitination of Ring1B. What is the role of these atypical chains? Surprisingly, when they tested H2A monoubiquitination using Ub mutants they found that the same lysine residues were necessary. To demonstrate that self-polyubiquitination of Ring1B is required for H2A monoubiquitination, the authors used an approach very similar to the one used by Woelk *et al*. Ring1B was first subjected to a self-ubiquitination reaction with wt or lysine-less Ub and then purified and used to perform H2A monoubiquitination. In agreement with the idea that self-ubiquitination of Ring1B should occur prior or simultaneously with ubiquitination of the substrate, the lysine-less Ub-modified Ring1B showed a significant reduction in its activity toward H2A [30].

Unfortunately, the currently available tools do not allow the detailed structure of the atypical chains generated by Ring1B to be defined. Likely, they represent mixed branched chains in which any Ub within the chain can be conjugated with several others via different internal lysines. The molecular function of the atypical mixed chains attached to Ring1B is not clear yet, but it is tempting to speculate that the Ub chains might alter the structure of the E3, rendering it more active, or, as in the case of Nedd4, changing its binding specificity. Whatever the case, whether and how ubiquitination of E3 ligases is regulated *in vivo *remains to be established.

**Figure 4:**
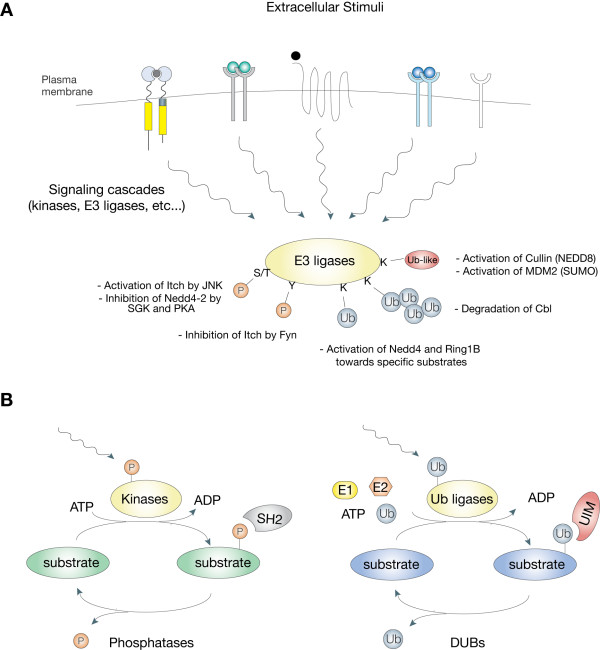
**Regulation of E3 activities by post-translational modifications**. A) Extracellular stimuli can induce various post-translational modifications of E3 ligases. This might result on a positive or a negative regulation of the ligase activity. Examples are reported (see main text for detailed explanations). The arrows represent the integration of numerous signals. pY, tyrosine phosphorylation; pS/T, serine or threonine phosphorylation; Ub, ubiquitination; Ub-like, neddylation or sumoylation. B) Comparison between the phospho-based network and the Ub-based network. Similarity includes recognition by dedicated protein domains (SH2 and UIM are example), inducibility by upstream signals and reversibility (DUBs work as phosphatases). In addition, kinases are often activated by tyrosine phosphorylation in the signalling cascade while E3 ligases could be activated by ubiquitination.

The fact that two totally unrelated E3 ligases, one of the HECT-type and the other of the RING-type, both appear to be regulated by ubiquitination, might indicate a more general mechanism of regulation and suggest another unexpected similarity between phosphorylation and ubiquitination pathways. In the same way that kinases are often activated by tyrosine phosphorylation in the signalling cascade, E3 ligases appear to be activated by ubiquitination (Figure 4).

Finally, Ub E3 ligases can be modified by ubiquitin-like modifiers, as well (Figure 4). Neddylation of cullin scaffold family members strongly activate the E3 activity of cullin-based ubiquitin E3s, such as SCFs [96,97]. Mdm2 increases its activity towards p53 upon sumoylation, while desumoylation increases Mdm2 self-ubiquitination and degradation, thereby stabilizing p53 [98].

## Conclusions and perspectives

Ubiquitin modification irreversibly or reversibly changes the fate of a target protein. Ubiquitination is regulated at different levels by the enzymes that catalyse the reaction, the properties of the substrate, the presence of UBDs or the activity of the enzymes that reverse the reaction, the DUBs. All these hardware components are frequently and rapidly modulated by extracellular signals (for example, growth factors, pro-inflammatory cytokines and UV damage) in a signalling cascade of ubiquitination events that mirrors the well-characterized phosphorylation cascade. Moreover, the two pathways are strongly interlinked. We have described how the phosphorylation of substrates or E3 ligases is, in some cases, a prerequisite for subsequent ubiquitination. We can hypothesize that the opposite might also occur as the presence of a UIM and of a RING domain in the MEKK1 enzyme seems to suggest. This additional layer of complexity is further amplified by a number of ubiquitin-like modifications present in the cell, not even discussed in the present review. We are only just starting to understand the basis of the system. It is predictable that the coming, exciting years will provide considerable insight into the regulation of diverse pathways, including cell proliferation and apoptosis, for which ubiquitination is of paramount importance. Uncovering the basic mechanisms in the ubiquitination system will help us to understand how their subversion contributes to diseases, such as cancer, and will provide a new window of opportunity for the development of novel therapeutic strategies of intervention.

## Competing interests statement

The author(s) declare that they have no competing interests.
